# Segmentation of Renal Thyroid Follicle Colloid in Common Carp: Insights into Perfluorooctanoic Acid-Induced Morphometric Alterations

**DOI:** 10.3390/toxics12050369

**Published:** 2024-05-17

**Authors:** Maurizio Manera, Luisa Giari

**Affiliations:** 1 Department of Biosciences, Food and Environmental Technologies, University of Teramo, St. R. Balzarini 1, 64100 Teramo, Italy; 2 Department of Environmental and Prevention Sciences, University of Ferrara, St. L. Borsari 46, 44121 Ferrara, Italy; luisa.giari@unife.it

**Keywords:** goitrogenic effect, folliculogenesis, endocrine disruptors, animal model, kidney, environmentally relevant concentration, image analysis, shape descriptor, one health

## Abstract

Perfluorooctanoic acid (PFOA) is a globally prevalent contaminant of concern recognised for its persistence and detrimental effects on both wildlife and humans. While PFOA has been established as a disruptor of thyroid function, limited data exist regarding its impact on thyroid morphology. The kidney of the common carp (*Cyprinus carpio*) harbours numerous thyroid follicles, rendering it a valuable biomarker organ for investigating PFOA-induced thyroid alterations. Renal tissue slides, stained with the Alcian blue/PAS method, were examined from carp in three experimental groups: unexposed, exposed to 200 ng L^−1^, and exposed to 2 mg L^−1^ of PFOA over 56 days. Thyroid follicle colloids were segmented, and related morphometric parameters, including perimeter, area, and shape descriptors, were obtained. Statistical analyses revealed significant reductions in thyroid follicle colloid perimeter and area in the 200 ng L^−1^ PFOA group compared to the unexposed and 2 mg L^−1^ PFOA groups. Additionally, the fish exposed to PFOA exhibited a significantly higher follicle count compared to the unexposed fish. These findings collectively suggest that PFOA induces thyroid folliculogenesis, emphasising its impact on thyroid morphology even at an environmentally relevant concentration (200 ng L^−1^).

## 1. Introduction

Perfluorooctanoic acid (PFOA) emerges as a prominent member within the expansive group of per- and polyfluoroalkyl substances (PFASs). PFOA’s molecular structure comprises a chain of seven fluorinated carbon atoms coupled with a carboxyl group, rendering it extraordinarily resistant to degradation due to robust carbon–fluorine (C-F) bonds and demonstrating exceptional surfactant properties [1]. In the mid-1900s, PFOA began to be produced and included in many industrial and commercial products, such as textiles, cookware, and food packaging [1]. PFOA captured scientific and public attention in the 2000s due to its ubiquitous presence across environmental matrices, including biota [2], and its implication in severe human contamination events [3,4].

Though some aspects of PFOA toxicity remain not fully elucidated, the past two decades have witnessed a surge in studies. This increased knowledge of PFOA has led to a high level of concern, recognition of its harmfulness (PFOA categorization as a Group 1 carcinogen by the International Agency for Research on Cancer [5]), and legal restrictions or bans on its usage in many countries [6,7]. 

Given its ubiquitous occurrence, high environmental persistence, and long half-life within organisms, PFOA continues to pose a significant health hazard [8]. Hence, elucidating its adverse effects and mode of action remains imperative, not only for risk assessment but also for comparative purposes in evaluating the safety of new emerging PFASs, which are replacing old ones such as PFOA [8,9,10]. The spectrum of health impacts attributed to PFOA exposure encompasses liver toxicity, immunotoxicity, certain cancers, endocrine system disruption, and alterations of reproduction and development [10,11,12]. 

Aquatic ecosystems are typical sinks and ways of transport and diffusion for PFOA, with aquatic organisms enduring prolonged or lifelong exposures, potentially leading to bioaccumulation [13,14]. Fish serve as pivotal subjects for PFAS research, functioning as biomonitoring agents, vectors for human contamination, and vertebrate models aligning with the One Health paradigm [15], and cyprinids have proven instrumental in delineating PFOA toxicity and endocrine disruption [13,16,17,18].

PFOA’s interaction with the thyroid gland has been evidenced across fish, mammals, and birds [10,19,20]. Given the evolutionary conservation of thyroid hormones (THs) across vertebrates and their pivotal roles in metabolic regulation, development, and growth, PFOA poses a tangible risk to both human and wildlife populations [10,20]. The impairment of the thyroid system by endocrine disruptors (EDs) such as PFOA encompasses various mechanisms that could act on thyroid gland components (for example, the modification of epithelium and/or colloid, cytotoxicity towards thyroid cells), receptors, transport proteins, and enzymes involved in TH metabolism [21]. Structurally akin to the hormone thyroxine (T4), PFOA and other PFASs can compete for binding to thyroid hormone receptors and carriers (e.g., transthyretin), thereby interfering with thyroid function [22,23,24]. Moreover, PFOA exerts modulatory effects on numerous thyroid-related genes [25,26,27,28]. Circulating TH levels could undergo alterations following PFOA exposure, generally exhibiting a decrease, although the available data present some controversy and variability depending on the species and PFOA concentrations [29]. Reduced concentrations of triiodothyronine (T3) and/or T4 have been observed in PFOA-treated zebrafish larvae [30], river rainbowfish experimentally exposed to four concentrations of PFOA [20], and adult perch naturally exposed to PFASs [31].

The present research focuses on common carp (*Cyprinus carpio*) and leverages the presence of numerous thyroid follicles in the renal tissues of this species [32]. This physiological characteristic renders the carp kidney an ideal organ for documenting PFOA-induced alterations to the thyroid system, as evidenced by previous work by Manera et al. [33], which reported increased abundance and structural/ultrastructural modifications of thyroid follicles in the kidneys of PFOA-exposed *C. carpio*.

While most studies investigating fish thyroid responses to PFASs primarily concentrate on hormone levels, enzyme activity, and gene expression modifications, Miranda et al. [20] advocate for the importance of also considering morphological effects to enhance our understanding of PFOA’s thyroid-disrupting potential. Among the limited reports based on histology, Ji et al. [34] and Lee et al. [35] conducted PFOA exposure tests on Medaka *Oryzias latipes* and documented various pathological changes in thyroid follicles in the offspring, indicating multigenerational consequences. The thyroid-disrupting effects of PFOA or other long-chain PFASs during development have been associated with delayed and abnormal developmental processes/events, decreased body dimensions, and reduced egg production in zebrafish [36].

Given the limited understanding of PFASs’ impact on thyroid morphology, the principal objectives of the present study are (i) to investigate the potential for PFOA to perturb the structure of thyroid follicles in carp under experimental exposure conditions and (ii) to evaluate a method for the morphometric assessment of thyroid follicle condition/status.

The current research highlights the susceptibility of carp thyroid follicles to PFOA exposure, even at an environmentally relevant concentration, with significant morphometric alterations (particularly a decrease in colloid area) and the advantages (i.e., reliability, repeatability, and ease of execution) of the colloid segmentation method with potential applications in toxicologic pathology and ED research. The increased number and diminished dimension of thyroid follicles observed in the exposed fish, along with alterations in shape descriptors, prompt the hypothesis that PFOA induces the de novo formation of thyroid follicles in carp kidneys. This folliculogenesis occurs in distinct patterns depending on the concentration of PFOA exposure.

## 2. Materials and Methods

The renal tissue slides utilised in this study were obtained from archive material originating from a previous experiment [37]. Readers are referred to this reference [37] for detailed information regarding the experimental design. Importantly, no fish were intentionally sacrificed for the purposes of the present study, aligning with ethical considerations.

### 2.1. Experimental Design Derived from Previous Research [37]

In brief, in this study, renal tissue slides from 15 two-year-old common carp (total length: 19.32 ± 2.49 cm; body mass: 104.84 ± 27.80 g [mean ± standard deviation]) from previous research [37] were examined. Of these, 5 slides were derived from an unexposed (control) group, while another 5 originated from a group exposed to 200 ng L^−1^ PFOA, and the remaining 5 from a group exposed to 2 mg L^−1^ PFOA for a duration of 56 days, constituting sub-chronic exposure. The exposure experiment was conducted in a flow-through open system with a water flow rate of 500 mL min^−1^ [37]. The chosen concentrations were selected based on specific criteria. The lower concentration (200 ng L^−1^ PFOA) was determined to mirror reported levels of perfluorooctanoic acid (PFOA) found in surface water [38], thereby representing ecologically relevant conditions. Meanwhile, the higher concentration (2 mg L^−1^ PFOA) was chosen based on previous studies, which demonstrated its capability to induce histological changes in cyprinid fish [39].

### 2.2. Tissue Processing for Light Microscopy from Previous Research [37]

The tissue slides used in this study were initially derived from kidney samples that were fixed in 10% neutral buffered formalin [37]. Subsequently, these samples underwent routine processing for light microscopy with paraffin embedding, sectioning performed at a thickness of 5 μm, and staining carried out using the Alcian blue 8 GX (pH 2.5)/periodic acid–Schiff’s reagent (PAS) method [37].

For the specific requirements of this investigation, a total of 15 renal tissue sections were examined and photographed using a Nikon Eclipse 80i light microscope (Nikon, Tokyo, Japan). These tissue sections comprised 5 sections from 5 unexposed fish, 5 sections from 5 fish exposed to 200 ng L^−1^ PFOA, and 5 sections from 5 fish exposed to 2 mg L^−1^ PFOA. Each tissue section was carefully examined under 200X magnification, following a zigzag trajectory to ensure comprehensive coverage. All microscopic fields containing thyroid follicles were photographed. The captured images were saved in TIF format with dimensions of 1440 × 1024 pixels, providing sufficient resolution (1 pixel = 0.52 μm) to grant accuracy during the segmentation process.

### 2.3. Thyroid Follicle Colloid Segmentation

The segmentation process prioritised thyroid follicle colloid due to its higher contrast with PAS staining, a commonly used staining method in histopathology and possibly more readily available in archive materials than colloid-dedicated staining methods. The segmentation of the entire follicle would have required complex algorithms and extensive computational processing [40], which were beyond the scope of the study. The focus was on identifying the most feasible method for estimating thyroid follicle morphometrics to evaluate the effect of PFOA exposure, potentially utilising archive materials. While acknowledging potential disparities between colloidal and true follicle (colloid plus lining epithelium) morphometrics, it was anticipated that the measurements obtained from colloids would provide meaningful insights, particularly as colloids closely correspond to the luminal surface of the follicular epithelium, the thyroid’s crucial functional interface [33]. Therefore, focusing on colloids allows for a more direct assessment of this morphofunctional interface. 

Thyroid follicle colloid segmentation was conducted using Orbit Image Analysis software (version 3.64, Actelion Pharmaceuticals Ltd., Allschwil, Switzerland), which operates under the GPLv3 license. The segmentation process followed the manufacturer’s instructions for object segmentation [41]. In brief, two classes were defined: the thyroid follicle colloid and the background, representing the rest of the tissue. The manual delineation of these regions was performed on representative images from all experimental groups to train the classifier. Subsequently, the classifier was applied to each image, and adjustments were made to segmentation settings through the feature configuration tab, following the manufacturer’s guidelines [41], where necessary.

As a result of segmentation, the following morphometric parameters were obtained for each single thyroid follicle colloid: perimeter (pixels), area (pixels^2^), circularity, roundness, convexity, compactness, and solidity (dimensionless). Shape descriptors were defined as follows:

- Circularity measures how closely an object’s shape resembles a perfect circle by comparing its area to its perimeter squared. A circularity value of 1 indicates a perfect circle, while values nearing 0 suggest elongated or irregular shapes. It is defined as follows: $circularity=\frac{4\times area\times \pi }{{perimeter}^{2}}$ [41].
- Roundness quantifies how closely an object’s boundary resembles a perfect circle by comparing its area to the square of its major diameter. Higher roundness values indicate shapes that are more circular, while lower values suggest irregular or angular shapes. It is defined as follows: $roundness=\frac{4\times area}{\pi \times {\max diameter}^{2}}$, where the max diameter is the major diameter of the object [41].
- Convexity measures the degree to which a shape bulges outward or is convex by comparing the perimeter of the object’s convex hull (the smallest convex shape enclosing the object) to its own perimeter. Convexity values range from 0 to 1, with higher values indicating smoother shapes. It is defined as follows: $convexity=\frac{convex\;perimeter}{perimeter}$, where the convex perimeter is the perimeter of the imaginary convex hull drawn around the object [41].
- Compactness provides a numerical value for how “round” or “squished” a shape is, somewhat similar to roundness but using a different scale factor. It is defined as follows: $compactness=\frac{\sqrt{\frac{4\times area}{\pi }}}{max\;diameter}$, where the max diameter is the major diameter of the object [41].
- Solidity assesses the proportion of the object’s area covered by its convex hull, indicating the solidity of the shape. Solidity values range from 0 to 1, with higher values indicating shapes that are more solid or filled in, while lower values suggest shapes with concavities on the surface. It is defined as follows: $solidity=\frac{area}{convex\;area}$, where the convex area is the area of the imaginary convex hull drawn around the object [41].

### 2.4. Statistical Analysis

The obtained numerical data underwent preliminary checks for the normality assumption and variance homogeneity using the Shapiro–Wilk and Levene’s tests, respectively. Since the data did not meet the assumption of a normal distribution, the Kruskal–Wallis test was chosen to examine potential differences among the experimental groups. Subsequently, post hoc comparisons were conducted using the Dunn pairwise test. Furthermore, the total number of thyroid follicle colloids detected in the tissue slides was examined for significant differences among the experimental groups utilising an ANOVA, given the normal distribution of the data. Statistical analyses were performed using JAMOVI Desktop (version 2.5.3 release, JAMOVI project).

## 3. Results

### 3.1. Thyroid Follicle Histology

**Figure 1:**
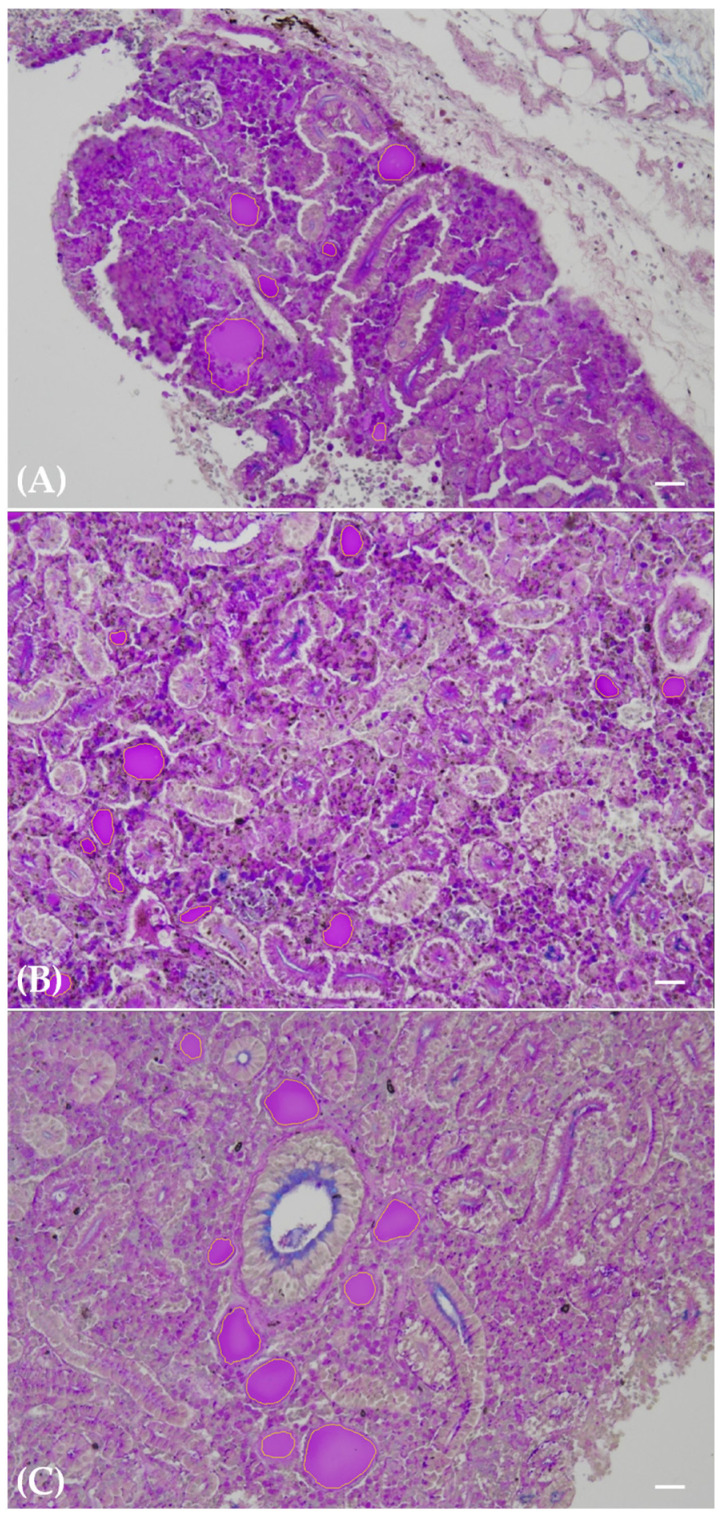
Paraffin-embedded tissue sections of carp kidneys: unexposed (**A**), exposed to 200 ng L^−1^ PFOA (**B**), and exposed to 2 mg L^−1^ PFOA (**C**). Alcian blue–PAS. Scale bar = 25 μm. Thyroid follicles are clearly visible in cross-sections as circular to ellipsoidal structures, varying in number and size according to the exposure group. The PAS positivity of the colloid enhances the segmentation process, delineating the follicular lumen with a yellow line. Particularly evident is the higher number and smaller cross-sectional area of thyroid follicles in the 200 ng L^−1^ PFOA exposure group (**B**) compared to the unexposed group (**A**).

The thyroid follicles were observed in cross-sections as circular to ellipsoidal structures, characterised by a lining of either flattened pavement or cuboidal cells enclosing a PAS-positive colloid-filled follicular lumen (Figure 1A–C). These follicles were dispersed within the renal hematopoietic tissue, occurring either as solitary units or clusters, with variations in both quantity and dimensions depending on the specific experimental exposure class (Figure 1A–C). Specifically, exposure to PFOA resulted in a heightened probability of observing multiple thyroid follicles within the same histological field. Furthermore, the follicles from the PFOA-exposed fish demonstrated reduced dimensions on average, with this effect notably pronounced in the experimental group exposed to 200 ng L^−1^ PFOA (Figure 1A–C).

### 3.2. Thyroid Follicle Colloid Segmentation

**Figure 2:**
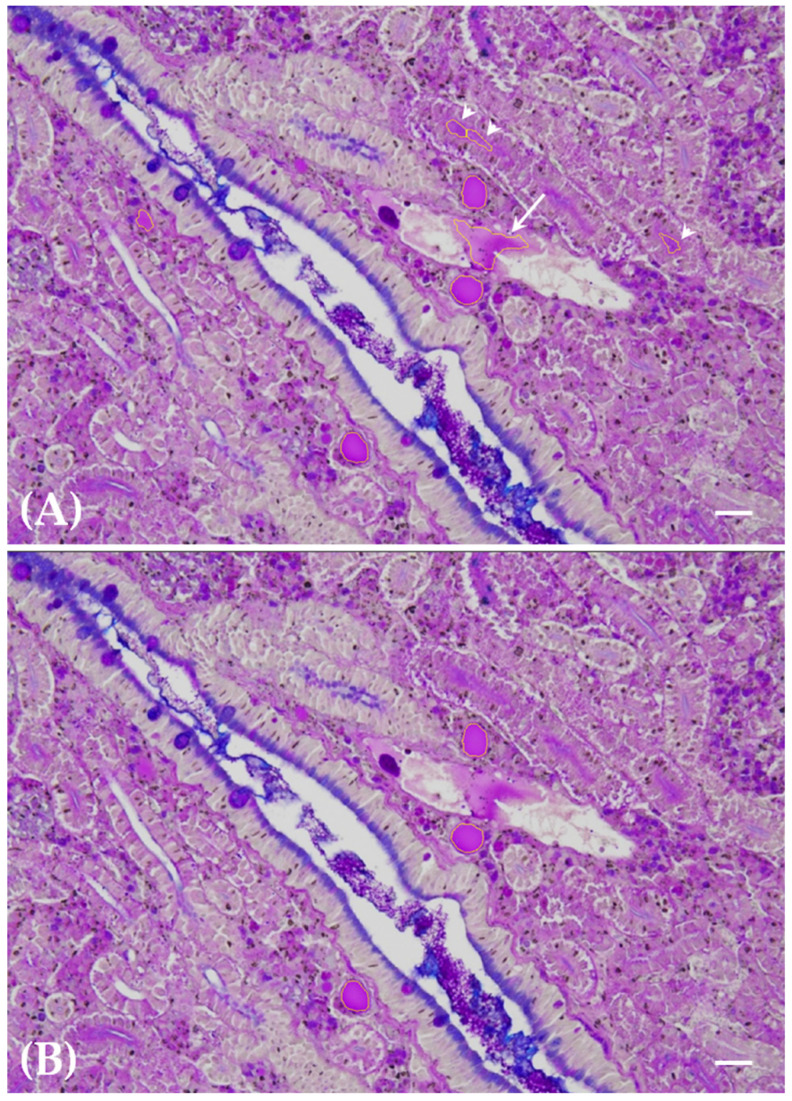
Paraffin-embedded tissue sections of carp kidneys from fish exposed to 200 ng L^−1^ PFOA. Alcian blue–PAS. Scale bar = 25 μm. Segmentation results before (**A**) and after (**B**) fine-tuning of segmentation parameters are depicted. Examples of falsely positively segmented structures, such as plasma in a vessel (arrow) and renal tubule brush border (arrowheads), due to their PAS positivity, are shown in panel (**A**). These structures were no longer visible after fine-tuning, leaving only correctly classified thyroid follicle colloids outlined by yellow lines in panel (**B**).

**Figure 3:**
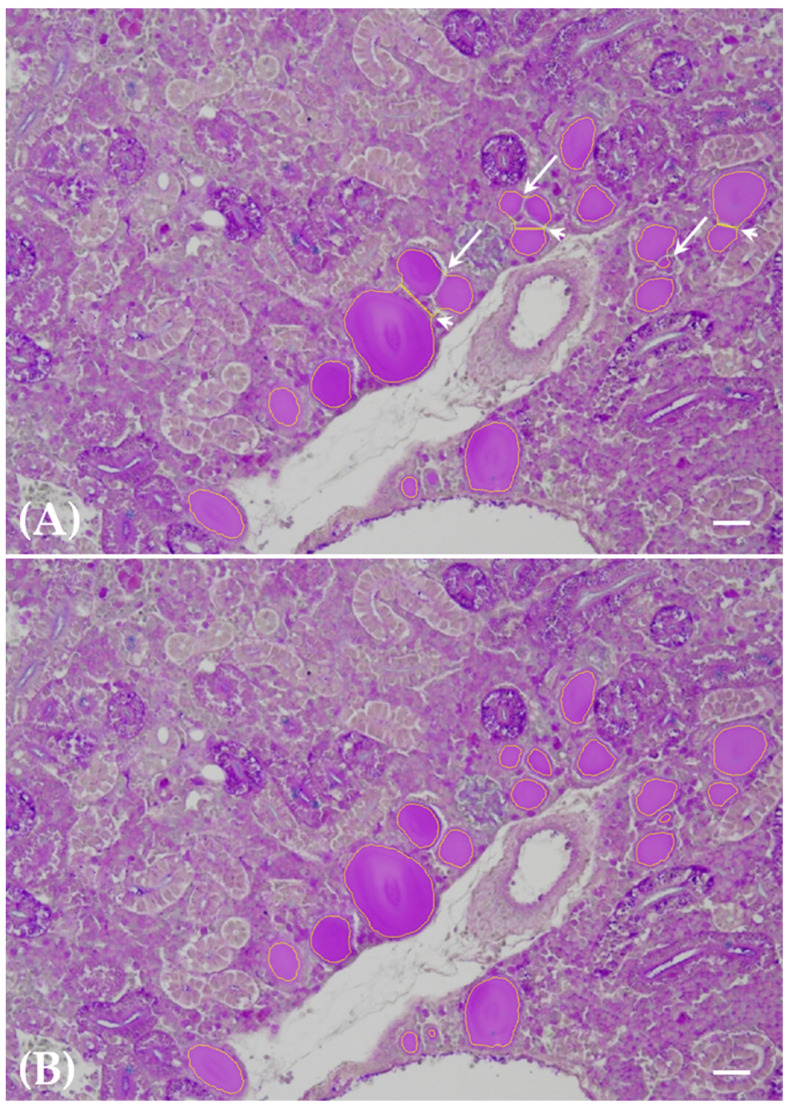
Paraffin-embedded tissue sections of carp kidneys from fish exposed to 2 mg L^−1^ PFOA. Alcian blue–PAS. Scale bar = 25 μm. Segmentation results before (**A**) and after (**B**) fine-tuning of segmentation parameters are depicted. Examples of falsely negatively segmented follicle colloids, attributed to the fusion of contiguous follicles (arrows) and the distortion of segmented forms resulting from the adhesion of contiguous follicles (arrowheads), are illustrated in panel (**A**). These biases were no longer apparent after fine-tuning, leaving only accurately classified thyroid follicle colloids outlined by yellow lines in panel (**B**).

Segmentation yielded a dependable identification of all thyroid follicle colloids within the renal tissue (Figure 1A–C), with no falsely segmented follicle colloids remaining after meticulous fine-tuning of the method. Conversely, a negligible 3% of follicle colloids persisted in being falsely classified as negative among the total number of segmented follicle colloids. These falsely unsegmented follicle colloids were primarily small entities, predominantly associated with the 200 ng L^−1^ PFOA exposure group. The outcomes of meticulous fine-tuning are illustrated in terms of false positive and false negative correction, as depicted in Figure 2A,B and Figure 3A,B, respectively.

### 3.3. Thyroid Follicle Colloid Morphometrics

**Figure 4:**
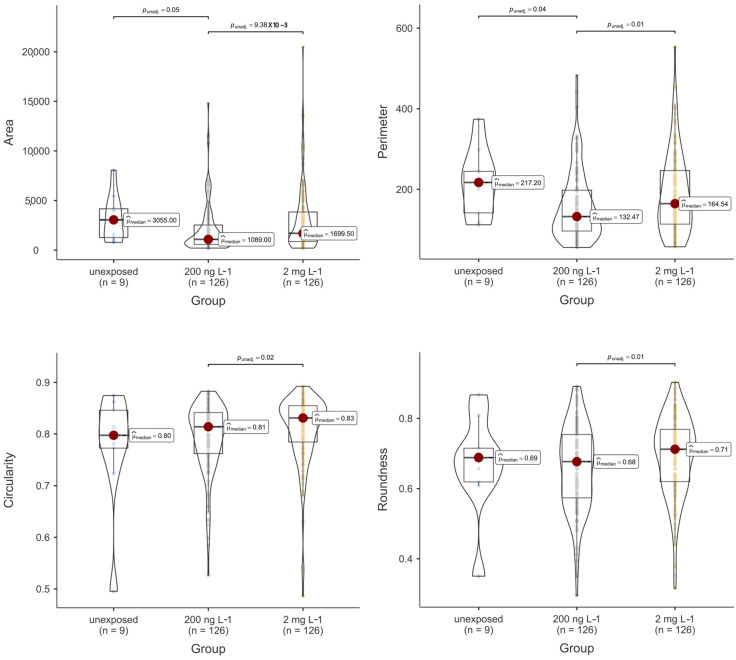
Violin and box plot of thyroid follicle colloids’ cross-sectional area (pixel^2^), perimeter (pixel), circularity, and roundness (dimensionless) according to experimental exposure group (*n* = number of screened thyroid follicle colloids). Each coloured bullet denotes a single data value for each morphometric parameter/experimental group. Significant differences, where present, are indicated by horizontal whiskers along with the respective *p*-values.

**Figure 5:**
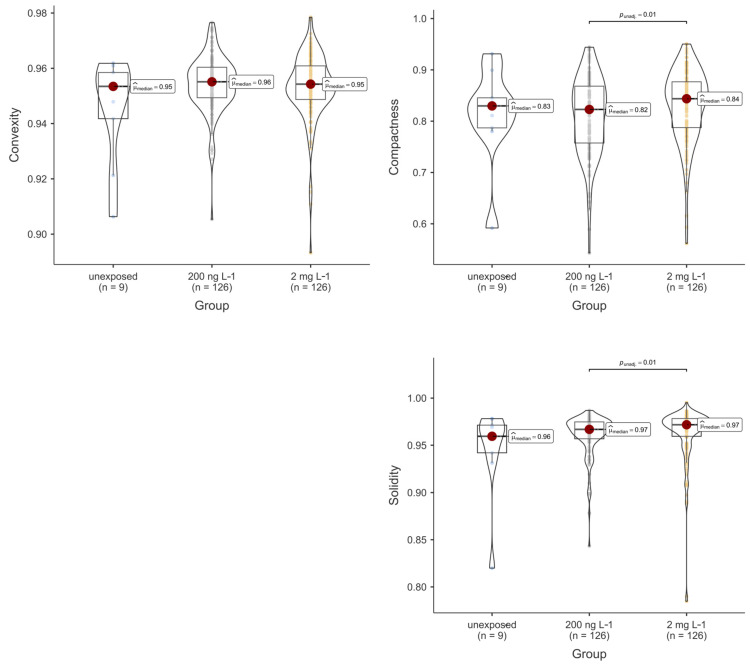
Violin and box plot of thyroid follicle colloids’ convexity, compactness, and solidity (dimensionless) according to the experimental exposure group (*n* = number of screened thyroid follicle colloids). Each coloured bullet denotes a single data value for each morphometric parameter/experimental group. Significant differences, where present, are indicated by horizontal whiskers along with the respective *p*-values.

**Figure 6:**
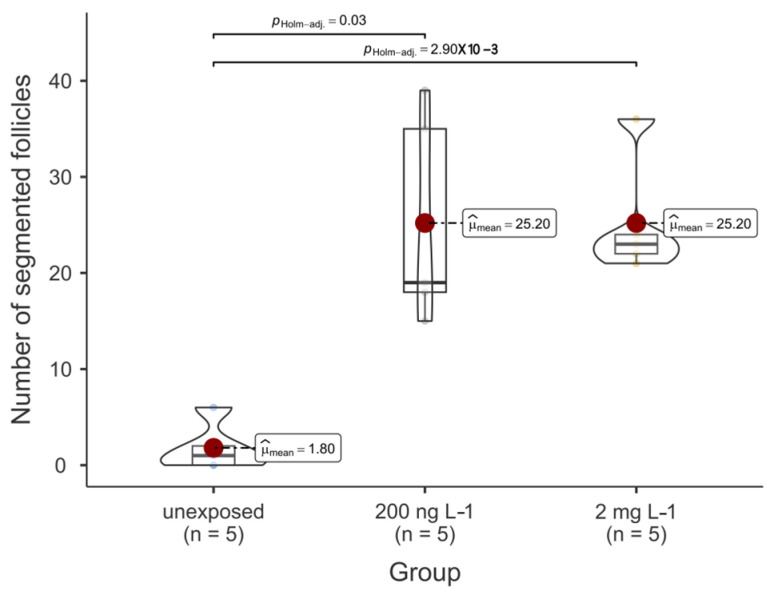
Violin and box plot of the number of segmented thyroid follicle colloids according to the experimental exposure group (*n* = number of screened tissue slides). Each coloured bullet denotes a single data value for each experimental group. Significant differences, where present, are indicated by horizontal whiskers along with the respective *p*-values.

The morphometric results of the thyroid follicle colloids are summarised in Figure 4 and Figure 5 and Table 1. Notably, PFOA was observed to influence these outcomes. Specifically, both the area and perimeter of the colloids from the fish exposed to 200 ng L^−1^ PFOA were significantly reduced compared to those from both the unexposed fish and the fish from the 2 mg L^−1^ PFOA exposure group. It is noteworthy that although the median of the area and perimeter of the thyroid follicles from the fish exposed to 2 mg L^−1^ PFOA appeared lower than that from the follicles of the unexposed fish, no significant difference was observed at the predetermined alpha value of 0.05. Regarding shape descriptors, significant differences were noted for all descriptors except convexity, with follicles from the fish exposed to 200 ng L^−1^ PFOA exhibiting lower values than those from the fish exposed to 2 mg L^−1^ PFOA. Practically speaking, the thyroid follicles exposed to 200 ng L^−1^ PFOA exhibited a significantly altered morphology, characterised by reduced roundness and increased indentations, in comparison to the follicles from the fish exposed to 2 mg L^−1^ PFOA. Furthermore, exposure to PFOA influenced the total number of thyroid follicle colloids detected in the tissue slides from fish across the various experimental groups, with the fish from both PFOA-exposed groups exhibiting a significantly higher (*p* < 0.01) colloid count compared to those from the unexposed group (Figure 6).

## 4. Discussion

The proposed segmentation method for thyroid follicle colloids, employing freely available open-source software and a commonly used histochemical staining technique in histopathology, resulted in a reliable, objective, and repeatable approach for accurately identifying and quantifying the key morphometric parameters of the follicular lumen outline in the common carp kidney. This method revealed significant effects of PFOA exposure on perimeter and area measurements, as well as the majority of the tested shape descriptors. Another study in the literature, conducted by different authors [32], attempted a morphological data analysis of colloids in cross-sectioned common carp kidney thyroid follicles. However, these authors relied on non-freely available commercial image analysis software and a staining technique specifically designed to enhance colloid identification, which is not commonly employed in histopathology. Unfortunately, the authors [32] of the aforementioned study did not provide detailed segmentation results, making it difficult to directly compare the reliability of their findings with those of the present research.

As stated in the Materials and Methods section, the decision to focus on thyroid follicle colloids rather than the entire follicle (epithelium and colloid) was based on practical and morphofunctional considerations. Practically, due to the higher contrast of a colloid, segmenting it is a relatively easier task compared to the entire follicle, which would require dedicated algorithms and heavy computational processing [40]. Morphofunctionally, the outline of colloids corresponds closely to the luminal surface of the follicular epithelium, the most crucial functional interface of the thyroid [33]. Consequently, there may be disparities when comparing colloidal morphometrics with those of true follicles. Nonetheless, in terms of order of magnitude, the measurements should be comparable, provided there are no drastic variations in the follicular lining epithelium and/or the colloids do not have very small cross-sectional axes. This is particularly relevant when the ratio between the colloid axes and the height of the follicular lining epithelium is near or lower than 1, as observed in species with compact thyroids presenting with parenchymatous goitre. This is an alternative to colloid goitre, where typical large colloid-filled follicles predominate [42,43,44]. To facilitate a comparative analysis, Table 2 includes morphometric data obtained or calculated from other studies, with an emphasis on the data from Geven et al. (2007) [32], which are procedurally comparable to the present research, as both studies utilised thyroid colloid segmentation by image analysis software. However, it is important to note that the data from Geven et al. (2007) [32], similar to the present research, were not normally distributed. Interestingly, the authors relied on the mode as a descriptive statistic [32] rather than the median value commonly used in non-normally distributed data [45], as adopted in the present research. Upon reviewing Table 2, it becomes apparent that aside from differences possibly arising from procedural aspects, other sources of variability in thyroid follicle morphometrics may exist, including sex, age, and dimension/body mass [32,46,47]. To ensure experimental reproducibility, it is crucial to strictly control these factors. This highlights the necessity of establishing a proper control experimental group rather than relying solely on reference values. In this respect, it is critical to note that one of the objectives of this study was to introduce a dependable methodology to assist operators in comparing samples to a defined control, thereby enabling the objective and reliable assessment of morphometric changes in thyroid follicular colloids caused by exposure to a recognised environmental contaminant. This study did not aim to determine reference morphometric values for thyroid follicular colloids using a wholly unsupervised method.

Based on the findings of this study regarding the dimensions of thyroid follicle colloids and the significantly higher colloid count observed in tissue sections from the PFOA-exposed fish compared to the unexposed group, which is consistent with the results of a prior investigation on the same experimental cohort [33], it is reasonable to conclude that PFOA exposure induces the de novo formation of thyroid follicles in the kidneys of carp. Additionally, this study reveals new insights not reported in the previous investigation [33], which addressed the effect of PFOA exposure on the biometry, structure, and ultrastructure of renal thyroid follicles. The increased presence of small thyroid follicles in the fish exposed to PFOA, particularly in the 200 ng L^−1^ PFOA exposure group, suggests a process of thyroid folliculogenesis [48,49]. Specifically, exposure to 200 ng L^−1^ PFOA was observed to enhance the formation of small to medium-sized thyroid follicles, while exposure to 2 mg L^−1^ PFOA led to the development of relatively larger follicles compared to the previous PFOA exposure group. 

In their study, Toda et al. (2003) [50] delineated two distinct pathways of thyroid folliculogenesis within an experimental model utilising thyroid tissue fragments cultured in a three-dimensional collagen gel. These pathways were identified as “mother follicle-derived folliculogenesis”, characterised by stages such as “solid nest formation, budding, and lumen-dividing types”, and “isolated cell-derived folliculogenesis”, which involved the development of follicles from either “isolated single or clustered thyrocytes” [50]. In the current research, irrespective of the specific mode of thyroid folliculogenesis, a consistent outcome emerged in the form of a median reduction in follicle dimensions. However, beyond the direct impact of folliculogenesis on this reduction, it should be explored whether additional contributing factors associated with PFOA exposure play a role. Previous studies on fish from the same experimental cohort as the current study have highlighted enhanced colloid macropinocytosis and/or reduced thyroglobulin production as potential consequences of PFOA exposure [33]. Therefore, in interpreting the present findings, it is crucial to consider these mechanisms alongside the dynamics of folliculogenesis.

In the presence of a discrete thyroid organ, thyroid hyperplasia, resulting from disruptions in the feedback mechanism involving thyrotropin-releasing hormone and thyroid-stimulating hormone (TSH), is commonly referred to as goitre [51]. Notably, folliculogenesis, in the form of a lumen-dividing type, has been reported to occur in nodular goitre [50,52]. While goitre can affect fish species as well [43,53,54], its applicability to common carp requires further examination. In common carp, thyroid follicles are dispersed throughout the subpharyngeal region, the pronephros, and the mesonephros, with the latter exhibiting the highest numbers and greatest thyroid hormonal activity [32]. Consequently, while the term “goitre” aligns functionally with the condition observed in common carp, its morphological relevance may be less clear in cases of renal thyroid follicle hyperplasia due to folliculogenesis, as in the present study. 

With regard to the potential pathogenesis of PFOA-induced alterations, this area has been relatively underexplored, likely due to species-specific differences in PFAS metabolism [29]. In a previous study conducted on fish from the same experimental cohort as the present research [33], PFOA was found to impact thyroid structure and ultrastructure in several ways. It induced changes in thyroid follicles, including an increase in follicle abundance and vesiculation, the latter being indicative of hyperactivity and/or early degeneration, particularly evident in the fish exposed to 2 mg L^−1^ PFOA [33]. At the ultrastructural level, PFOA exposure resulted in increased cellular projections, enhanced colloid endocytosis, enlargement and fragmentation of the rough endoplasmic reticulum, and cytoplasmic vacuolation [33]. Collectively, these alterations were interpreted as indicators of disruption in both the exocrine and endocrine phases of thyroid hormone synthesis [33]. However, the subtle ultrastructural changes observed, particularly in the fish exposed to 200 ng L^−1^ PFOA [33], may indicate an effect of TSH stimulation on thyroid follicle cells. This phenomenon has been experimentally documented in vitro in bovine TSH-stimulated thyroid tissue obtained from Hawaiian parrotfish (*Scarus dubius*), which are considered a suitable fish model due to their possession of a discrete and compact thyroid [55]. In an experiment involving zebrafish (*Danio rerio*) embryos exposed to 250 μg L^−1^ of perfluorooctanesulfonic acid (PFOS), no evident morphological changes were observed under light microscopy, except for a reduction in the nuclear area of follicular epithelial cells [56]. However, at the ultrastructural level, the vacuolation of the mitochondria and an enlargement and degranulation of the rough endoplasmic reticulum were observed [56]. While these ultrastructural alterations were thought to contribute to thyroid functional impairment and a decline in thyroid hormone levels, no precise pathophysiological explanation was provided [56]. Interestingly, PFOA has been reported to disrupt thyroid function by impairing iodine uptake by thyroid cells, interfering with thyroglobulin synthesis, and modifying thyroperoxidase (TPO) activity, as assessed in an in vitro study on human cells [57]. Another study [31] investigated the hepatic mRNA expression of thyroid-related genes in perch (*Perca fluviatilis*) from PFAS-contaminated and reference lakes. The results showed a downregulation of type 2 deiodinase (dio2) expression, promoting the conversion of inactive T4 hormone to active T3, and an upregulation of type 3 deiodinase (dio3) expression, facilitating the inactivation of T3 and T4 in fish from the contaminated lake. This suggests that PFAS exposure may alter deiodinase activity, potentially leading to decreased plasma T3 levels in fish from the contaminated site [31]. Although the present survey did not include endocrinological or biomolecular testing of thyroid function, as it was based solely on archived material, it is noteworthy that previous investigations within the same experimental cohort examined the expression levels of the CYP19A gene [58]. This gene encodes the enzyme responsible for converting testosterone to oestrogen. In male and female carp exposed to either 200 ng L^−1^ or 2 mg L^−1^ of PFOA, alterations in gonadal tissues were observed, with increased expression in male gonads and decreased expression in female gonads. Notably, these changes occurred irrespective of PFOA concentration [58]. Thus, even at environmentally relevant concentrations (200 ng L^−1^), PFOA demonstrated its potential to act as an endocrine disruptor [58].

As a consequence, the following speculative pathophysiological hypothesis may be proposed: PFOA could impair thyroid hormone production through several non-mutually exclusive mechanisms:

- Impaired thyroglobulin synthesis;
- Impaired thyroglobulin iodination;
- Impaired colloid macropinocytosis and thyroglobulin clivage;
- Altered hepatic deiodinase activity.

These effects could lead to a reduction in active thyroid hormones, subsequently stimulating an increased production of TSH through feedback mechanisms [44,59]. Ultimately, this dysregulation may contribute to the observed thyroid follicle folliculogenesis, accompanied by a considerable reduction in colloid cross-sectional area [49,59] in the PFOA-exposed fish, particularly in the 200 ng L^−1^ PFOA exposure group.

The observed discrepancy, wherein the thyroid follicle colloids from the fish exposed to 200 ng L^−1^ PFOA exhibited a more pronounced reduction in colloid cross-sectional area compared to those exposed to 2 mg L^−1^ PFOA, raises questions. This is particularly noteworthy given the occurrence of falsely negative segmented thyroid follicle colloids (representing very small entities) exclusively in the 200 ng L^−1^ PFOA group, alongside significant differences in most of the tested shape descriptors among the PFOA-exposed groups. These findings prompt consideration of whether a distinct folliculogenesis pattern may have occurred. Specifically, there could be a prevalence of “isolated cell-derived folliculogenesis” [50] in the 200 ng L^−1^ PFOA group, resulting in relatively smaller, less rounded (lower circularity, roundness, and solidity), and less smooth (lower compactness) follicles, and “mother follicle-derived folliculogenesis” [50] in the 2 mg L^−1^ PFOA group yielding relatively larger, more rounded (higher circularity, roundness, and solidity), and smoother (higher compactness) follicles. This hypothesis suggests that exposure to different concentrations of PFOA may have elicited varying effects on the follicular development process, potentially leading to the observed disparities in morphometric parameters within the present study.

The possibility of cytotoxic effects at the highest tested concentration (2 mg L^−1^ PFOA) should be considered in the context of underlying pathogenesis. A cytotoxic effect was reported in a previous study from the same experimental set as the present, where ultrastructural signs suggestive of incipient degeneration were observed in fish exposed to 2 mg L^−1^ of PFOA [33]. Thyroid hormone regulation involves a complex network that extends beyond the traditional hypothalamus–pituitary–thyroid axis, encompassing other organs such as the liver and kidney [60,61]. Consequently, morphofunctional alterations in these organs can lead to disruptions in thyroid function [62,63,64]. Interestingly, previous studies have demonstrated that PFOA exposure affects the liver and kidney of fish from the current experimental set in a dose-dependent manner [65,66,67,68]. This finding suggests that the observed discrepancy may have also been influenced by the dose-dependent effects of PFOA on these organs.

The current investigation utilised archive material from a previous experiment [37], aligning with ethical considerations regarding animal welfare. However, this reliance on archived samples presents a limitation: thyroid follicle analysis occurred solely at the conclusion of the 56-day experimental exposure period, representing morphologically a consolidated state. Consequently, while signs of “isolated cell-derived folliculogenesis” [50] persisted in the 200 ng L^−1^ PFOA group, unfortunately, histological evidence of “mother follicle-derived folliculogenesis” [50] was undetectable. This highlights the need for future, targeted studies involving multiple subsequent sample collections during the exposure experiment to document the latter phenomenon, which can only be hypothesised at present. Further investigation into the cellular mechanisms underlying the observed differences, alongside targeted endocrinology and biomolecular research, is warranted to elucidate the potential role of PFOA in modulating folliculogenesis patterns.

## 5. Conclusions 

The study introduces a reliable method for segmenting thyroid follicle colloids in common carp kidneys, revealing the notable impacts of PFOA exposure, particularly at an environmentally relevant concentration of 200 ng L^−1^. These findings underscore the urgency of addressing PFOA contamination and demonstrate the effectiveness of the method in detecting subtle environmental pollutant effects. Furthermore, the study enhances our understanding of PFOA-induced thyroid pathophysiology, highlighting potential goitrogenic effects and the need for further investigation into underlying molecular mechanisms. Emphasising the importance of appropriate animal models in environmental toxicology research, the study supports the One Health approach, promoting the interconnectedness of human, animal, and environmental health. Overall, this research makes a substantial contribution to our understanding of PFOA toxicity and the development of dependable methods for addressing environmental toxicology concerns, thus enriching our knowledge in the realm of thyroid disruption studies.

## Figures and Tables

**Table 1:** Thyroid follicle colloids’ morphometrics, according to experimental exposure group *.

Descriptive Statistics	Group	Perimeter	Area	Circularity	Roundness	Convexity	Compactness	Solidity
Mean ± standard deviation	Unexposed	113 ± 45	885 ± 661	0.77 ± 0.12	0.67 ± 0.15	0.95 ± 0.02	0.81 ± 0.10	0.95 ± 0.05
	PFOA 200 ng L^−1^	82 ± 44	555 ± 655	0.80 ± 0.07	0.66 ± 0.12	0.95 ± 0.01	0.81 ± 0.08	0.96 ± 0.02
	PFOA 2 mg L^−1^	98 ± 52	798 ± 881	0.81 ± 0.07	0.69 ± 0.11	0.95 ± 0.01	0.83 ± 0.07	0.96 ± 0.03
Median	Unexposed	113	829	0.80	0.69	0.95	0.83	0.96
	PFOA 200 ng L^−1^	69	295	0.81	0.68	0.96	0.82	0.97
	PFOA 2 mg L^−1^	86	461	0.83	0.71	0.95	0.84	0.97

* Unexposed, *n* = 9; PFOA 200 ng L^−1^, *n* = 126; PFOA 2 mg L^−1^, *n* = 126. Perimeter (μm); area (μm^2^); circularity, roundness, convexity, compactness, and solidity (dimensionless).

**Table 2:** Thyroid follicles’ morphometric data obtained or calculated from other authors’ studies *.

Group/Follicle Type	Perimeter	Area	Circularity ^1^	Roundness ^1^	Reference	Note
-	125 ± 8	963 ± 91	0.88 ± 0.01	0.72 ± 0.02	[32]	Measures pertain to thyroid follicle colloids, as originally reported by the authors. The values represent average mode values ± s.d., based on a sample size (n) of 4. The measurements were conducted on fish from an all-male E4XR3R8 isogenic strain. The body mass of the specimens was reported as 39.3 ± 0.5 g.
Small follicles	110	962	-	-	[46]	Derived from the mean major and minor follicle axes initially reported by the authors, based on measurements from three carp specimens with body masses ranging from 500 to 690 g and body lengths between 32 and 36 cm.
Large follicles	552	21,936	0.90	0.63	[46]	
Small follicles/summer	126	1257	-	-	[47]	Derived from the mean follicle diameter originally reported by the authors. The measurements were obtained from 12 adult female carp specimens.
Large follicles/summer	270	5809	-	-	[47]	
Small follicles/winter	154	1886	-	-	[47]	Derived from the mean follicle diameter originally reported by the authors. The measurements were obtained from 12 adult female carp specimens.
Large follicles/winter	308	7543	-	-	[47]	

* Perimeter (μm); area (μm^2^); circularity; roundness (dimensionless). ^1^ Circularity and roundness values were calculated only if distinct major and minor follicle axis values were reported.

## Data Availability

The datasets used and/or analysed during the current study are available from the corresponding author upon reasonable request.
